# Understanding metabolite transport gives an upper hand in strain development

**DOI:** 10.1111/1751-7915.13347

**Published:** 2018-12-03

**Authors:** Irina Borodina

**Affiliations:** ^1^ The Novo Nordisk Foundation Center for Biosustainability Technical University of Denmark Kongens Lyngby Denmark

## Abstract

Cell factories can be engineered for more efficient product secretion through modulation of membrane transporters.

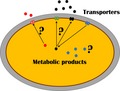

What if we knew how metabolites were transported inside and outside of cells? As a metabolic engineer, one could make cell factories export the product into the fermentation broth, likely improving the process performance and lowering the cost of downstream processing. If the product secreted from the cell, it would not inhibit its biosynthesis, it would be less toxic, and it would less likely be degraded. One could also prevent the leakage of pathway intermediates and improve the precursor supply in a specific cellular compartment.

Despite these apparent advantages, strain development programs often overlook metabolites transport. As an illustrative example, one can search for the occurrence of words ‘transport’ or ‘export’ among the papers published in Metabolic Engineering journal over the past five years, to discover that these words occur in only 6% of the papers (PubMed). One might wonder why? There may be at least three good reasons. First, there is a disagreement in the scientific community as to whether small metabolites are mainly transported through transporter proteins or if they also diffuse directly through the lipid bilayer (Sugano *et al*., 2010; Kell *et al*., 2011). Second, the methods for analysing transporter activity are technically complicated and require investment in specialized equipment and the necessary expertise (Brouwer *et al*., 2013). It is in contrast to biochemical methods for enzyme activity, where many assays can be implemented on a simple spectrophotometer. Third (and consequently), as the knowledge on transport remains limited, so are our abilities to modulate the transport processes. Hence, there are relatively few success stories on how such knowledge can be applied for strain development. These factors create a vicious circle, where limited knowledge and complicated methodologies discourage the scientists to enter this research area, and so it remains uncharted.

An eukaryotic model organism yeast *Saccharomyces cerevisiae* has 341 putative transporters predicted from its genome sequence (http://www.membranetransport.org). For the majority of the annotated transporters, the function was assigned based on sequence similarity and was not experimentally validated. A recent yeast genome‐scale metabolic model iIN800 contains ca. 220 unique transport reactions, which are physiologically essential or have been experimentally validated to occur in the cells (Nookaew *et al*., 2008). For less than half of these transport reactions, a possible responsible transporter is assigned. For the export of non‐native compounds, the responsible transporters are largely unknown.

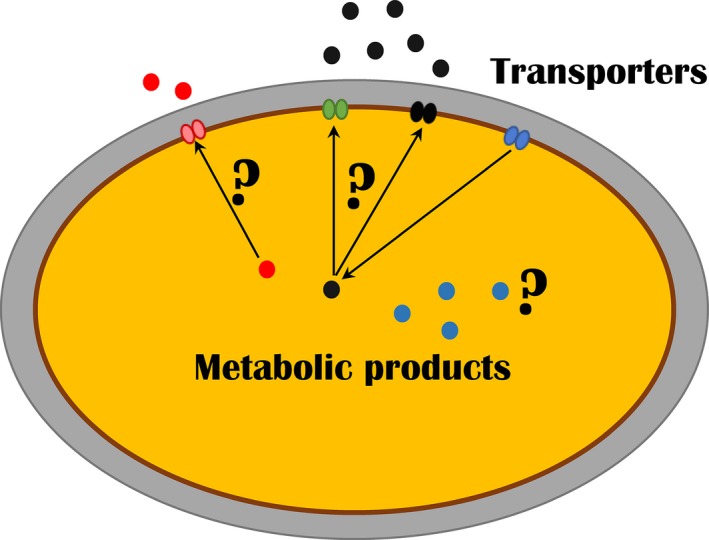

Nevertheless, several successful transport engineering cases inspire confidence that this is a promising research direction. The widely used flavour enhancer monosodium glutamate (MSG) has been produced by fermentation of coryneform bacteria since 1957 (Sano, 2009). The original process was based on wild isolates, which were capable of secreting several grams per litre of L‐glutamate under the right cultivation conditions. It was first recently discovered that the efficient secretion was due to a mechanosensitive channel, which released osmoprotectants, such as glutamate, in response to changes in membrane tension (Nakamura *et al*., 2007). *Corynebacterium glutamicum* is perhaps the best example of a cell factory, where the transporters have been successfully engineered to enable efficient production of multiple amino acids and derived products (Pérez‐García and Wendisch, 2018).

Another illustrative example is the engineering of dicarboxylic acid secretion by yeasts. While yeast *S. cerevisiae* is an attractive host for production of carboxylic acids due to its excellent tolerance to low pH, the transporters for commercially attractive endogenous (e.g., succinic, fumaric, malic) and exogenous (e.g., adipic, *cis*,*cis*‐muconic, 3‐hydroxypropionic) acids are unknown. Evolutionary, it cannot be expected that *S. cerevisiae* would naturally be efficient at secreting endogenous organic acids because of the high cellular demand for these metabolites. The same applies for exogenous metabolites; there is meagre chance that yeast would encode an efficient transporter for these metabolites as it neither produces nor utilizes these metabolites in nature. In contrast, natural producers of metabolites are likely to encode highly active efflux transporters for these compounds.

Overexpression of transporter‐encoding gene MAE1 from malate‐utilizing yeast *Schizosaccharomyces pombe* has dramatically improved the secretion of succinic and malic acids in *S. cerevisiae* and some other yeasts (Hara *et al*., 2017). We recently found that MAE1 most likely belongs to the family of slow anion channels (SLAC1) and not to proton‐ or sodium‐coupled transporters. The transporter thus operates with minimal energy use (our unpublished results). In general, energy usage of transporters should be taken into account when engineering cell factories; there seems to be an overall evolutionary trend towards energetically less costly transport that we described in (Darbani, 2018).

As the advantages of addressing the transport issue become more apparent, the transporter research area will grow and mature. New *in vitro* and *in vivo* methods will be developed for studying transporter specificity and kinetics; the throughput of these methodologies will be scaled up. We are currently building a whole transportome library of model eukaryote *Saccharomyces cerevisiae* for screening in *Xenopus* expression system and via solid surface membrane methodology (Nour‐Eldin *et al*., 2012; Bazzone *et al*., 2017).

In a few years, it will be possible to screen and identify exporters and importers of any desired metabolic product. This knowledge will enable engineering of the transport of metabolites, both in between the cellular compartments and outside the cell.

Transporter studies are essential not only for the metabolic engineering field but also for drug development. Drug transport may cause drug failure in clinical trials when the drug is poorly taken up in the target tissue/organ or is accumulated in non‐target organs. Multidisciplinary ongoing research efforts on deorphanizing the human transportome (e.g., https://re-solute.eu/) will contribute to the development of methods for transporter analysis and will improve the drug development pipeline.
